# Water, sanitation and hygiene in rural Greater Letaba Municipality, South Africa

**DOI:** 10.4102/hsag.v30i0.2940

**Published:** 2025-10-22

**Authors:** Mapula L. Molewa, Tobias G. Barnard, Nisha Naicker

**Affiliations:** 1 Water and Health Research Centre, Faculty of Health Sciences, University of Johannesburg, Johannesburg, South Africa; 2 Department of Environmental Health, Faculty of Health Sciences, University of Johannesburg, Johannesburg, South Africa; 3 Department of Epidemiology and Surveillance, National Institute for Occupational Health, National Health Laboratory Services, Johannesburg, South Africa

**Keywords:** sanitation, water, hygiene, waste management, diarrhoea, rural areas

## Abstract

**Background:**

Limited access to improved water, sanitation and hygiene (WASH) facilities disproportionately affects low- and middle-income nations, impacting human well-being, health, education and income.

**Aim:**

This study aimed to assess access to water, improved sanitation and hygiene practices as well as to describe the occurrence of diarrhoeal illnesses in the Greater Letaba Municipality (GLM) in Limpopo, South Africa.

**Setting:**

The study was conducted in the villages of Ward 2 of the Bolobedu region under GLM.

**Methods:**

A cross-sectional study sampled 120 households through a multistage probability sampling design. Data on WASH access and diarrhoeal episodes were collected through a pre-tested structured interview questionnaire and analysed with STATA 18.0.

**Results:**

Although all households had toilets, primarily pit latrines (92%), access to improved water sources was limited, with 62% depending on communal taps. Seventy-four per cent of households had access to handwashing facilities with soap and water. Eighty-one households (68%) reported that family members consistently washed their hands with soap and water after using the toilet. Most households (67%) experienced occasional diarrhoea over the past year. Diarrhoea prevalence was significantly correlated (*p* ≤ 0.001) with water storage practices, availability of handwashing facilities and the frequency of post-toilet handwashing.

**Conclusion:**

Despite universal toilet access, WASH infrastructure remained inadequate.

**Contribution:**

The study highlights the association between precarious water storage, handwashing practices and diarrhoeal illness, emphasising the importance of promoting hygiene alongside infrastructure development in rural areas.

## Introduction

Access to improved Water, Sanitation and Hygiene (WASH) facilities is not only a fundamental human right but also a critical factor influencing global health, well-being and socio-economic development (Nannan et al. 2022). Limited access to safe drinking water, proper sanitation and hygienic practices disproportionately affects low- and middle-income countries, playing a substantial role in poverty, impacting the health of women and children, as well as hindering education (Hothur, Arepalli & Doddoju Veera Bhadreshwara 2019). The World Health Organization (WHO) and the United Nations Children’s Fund (UNICEF) Joint Monitoring Programme for Water Supply and Sanitation (JMP) classify water sources as ‘improved’ when the water is delivered via piping into the residence, obtained via a manually operated pump, or derived from a safeguarded well (UNICEF & WHO 2023).

In contrast, ‘unimproved’ water sources are characterised by stagnant water from rivers, ponds or dams, as well as water collected and stored in rainwater tanks. Similarly, sanitation facilities are categorised as ‘improved’ if the excreta are safely disposed of in situ or removed from off-site (UNICEF & WHO 2023). Households lacking any latrine or toilet facility are considered to have ‘unimproved’ sanitation (Kassie & Hayelom 2017). Hygiene practices are deemed ‘poor’ if there are no handwashing or bathing facilities with detergents available in the household, or if hands are washed with only water and no soap (Wolf et al. 2023). In contrast, ‘good’ hygiene practices involve readily available handwashing and bathing facilities with soap and detergents (Kassie & Hayelom 2017; UNICEF & WHO 2023). In 2019, an estimated 1.4 million deaths (95% confidence interval [CI]: 1.3–1.5 million) and 74 million disability-adjusted life-years (DALYs) (95% CI: 68–80 million) could have been prevented through improved WASH. This accounts for 2.5% of all global deaths and 2.9% of global DALYs from all causes (Wolf et al. 2023). Approximately 7.75% (95% CI: 5.99–9.7) of all diarrhoeal-related deaths in sub-Saharan Africa are attributed to unsafe WASH practices. The corresponding risk factor attribution (RFA) is estimated at 95.93% (95% CI: 91.94–98.24), highlighting the substantial contribution of inadequate WASH conditions to the burden of diarrhoeal diseases (Zerbo, Castro Delgado & Arcos González 2021).

The importance of WASH in South Africa is underscored by a national risk assessment study: the South African Comparative Risk Assessment (SACRA1), which attributed a significant disease burden to unsafe WASH conditions. SACRA1 estimated that WASH-related factors ranked eleventh in terms of mortality and seventh in terms of DALYs among 17 evaluated risk factors (Bradshaw et al. 2022; Nannan et al. 2022). This equates to 2.4% to 2.7% of all deaths in South Africa, with a particularly severe impact on children under the age of five (Nannan et al. 2022). Insufficient WASH conditions represent a major public health risk, especially in developing nations (Okesanya et al. 2024; Wolf et al. 2023). It can leave the population vulnerable to a multitude of waterborne illnesses and can create a breeding ground for disease transmission (Wolf et al. 2023). Contaminated water serves as a reservoir for pathogens responsible for a range of illnesses, including dysentery, typhoid, polio and cholera (WHO 2022; Wolf et al. 2023).

The lack of proper sanitation facilities creates ideal conditions for the spread of a variety of diseases beyond diarrhoeal illnesses (Okesanya et al. 2024; Wolf et al. 2023). Just as contaminated water poses a health risk, poor sanitation also facilitates the transmission of diseases like cholera and schistosomiasis, as well as faecal-oral diseases like hepatitis A (Hothur et al. 2019; Wolf et al. 2023). Furthermore, inadequate waste disposal exacerbates the problem (Troeger et al. 2018). The buildup of organic waste and overflowing trash can contaminate the air, land and water. Also, it can attract pests that contribute to unsanitary conditions (Wang et al. 2019). Therefore, it significantly impacts public health and potentially spreads additional diseases. Conversely, good hygiene practices within households play a crucial role in controlling pest infestations. Maintaining a clean environment by eliminating food, water and harbourage sites for pests significantly reduces their populations (Gondhalekar et al. 2021; Wang et al. 2019). Apart from disease and pest prevention, the United Nations now recognises a healthy environment as a fundamental human right (United Nations General Assembly 2022).

The international community increasingly recognises that addressing the WASH crisis is essential to improve public health outcomes and promote human development (Wolf et al. 2023). By 2015, the chances of those without access to safe drinking water and acceptable sanitation are anticipated to drop by 50%, as per Millennium Development Goal 7 (MDG-7). The successor, Sustainable Development Goal (SDG), aims to ensure everyone has access to safe drinking water, adequate sanitation and hygiene by 2030 (Hothur et al. 2019; WHO/UNICEF JMP 2021; Wolf et al. 2023). Many people living in low-resource settings still lack access to these essential services. Reports on WASH practices, especially from rural areas of Limpopo Province, are limited. This study aimed to assess access to water, improved sanitation and hygiene, as well as to describe the occurrence of diarrhoeal illnesses in Ward 2, Greater Letaba Municipality (GLM).

## Research methods and design

### Study design

This cross-sectional study was conducted in March 2021 in rural Ward 2 of the GLM, Limpopo Province, South Africa, as part of a larger study titled ‘The assessment of the role of cockroaches in bacterial dissemination in relation to sanitary–hygienic conditions in ward 2 villages of Bolobedu area, Limpopo Province’.

### Setting

Ward 2 has a population of 5050 individuals residing in 1240 households across six villages: Mokwasele, Ramphenyang, Makaba, Mohlakong, Moshakga and Motsinoni (Greater Letaba Municipality 2022). The region features a challenging landscape with steep mountains. Households were defined by multigenerational living arrangements. A typical homestead comprised several huts clustered around a central courtyard. Farming activities were minimal, including growing crops and sometimes keeping livestock.

### Study population and sampling strategy

A sample size of 120 households was calculated using the Centres for Disease Control and Prevention (CDC)’s Epi Info 7.2 software for a descriptive survey. This estimate measured a 95% confidence level and a 5% margin of error, leading to a target of about 19.1 houses per village. To ensure proportional representation and randomisation, a multi-stage probability sampling design was employed as it allowed more flexibility given the geographical landscape, scattered households and lack of proper street demarcations in the study area. Twenty households were selected from each village in Ward 2. Trained research assistants were assigned to each village and instructed to select a random starting direction for movement. Every fifth residence encountered along the selected path was then incorporated into the study sample. A household is defined as a collective of individuals residing together and sharing meals. Only one participant per household was eligible to answer the questionnaire. Participants had to be at least 18 years old, and permanently residing in the selected household within the corresponding village of Ward 2 to provide accurate information. Individuals from the same household were not included separately.

### Data collection

Quantitative data were gathered through structured interviews. The questionnaire covered topics including household demographics, types of toilets, waste management practices, water sources, sanitation facilities, hygiene behaviours and past occurrences of diarrhoeal illness within the household. A preliminary pilot study preceded the main research period to test major study components. To validate the study design and instruments before full implementation, a pilot study was executed. This involved evaluating the pilot data to examine participant responses, the feasibility of the sampling strategy, the consistency of questionnaire administration by data collectors and the reliability of the questionnaire. The overarching goal was to preemptively address potential response, sampling, wording and contextual biases.

### Data analysis

Following data verification, all responses were transcribed into Microsoft Excel 2016. Data were cleaned and further analysed using STATA 18.0 software (StataCorp LLC, College Station, TX, US). Chi-squared tests were used to assess the statistical significance of results. Results were presented using basic frequency tables for ease of interpretation.

### Ethical considerations

Ethical approval for the study was obtained from the University of Johannesburg Faculty of Health Sciences (FHS) Research Ethics Committee (REC-866-2020), the Higher Degree Committee (HCD-01-105-2020), and the GLM. Prior to data collection, informed consent was obtained from each participant after a thorough explanation of the study’s purpose.

## Results

A total of 120 households participated in the study, achieving a 100% response rate with no dropouts after consent was obtained. The most common household size was five occupants (18%), followed by four occupants (15%) and three occupants (13%). Households with six and eight occupants each constituted 12%, while those with seven residents accounted for 9%. In addition, 7% of households had two occupants, and 3% had a single occupant. A very small proportion (1%) of households had as many as 13 individuals residing within them. The average number of residents per household was 5.67. Table 1 details the water sources, sanitation facilities and waste disposal strategies utilised by participating households, as reported by the study participants.

**TABLE 1:** Access to water, sanitation and waste disposal facilities.

Variables	Category	*n*	%
Primary source of water	Own borehole	12	8
	Communal tap	95	62
	Municipal mobile supply	14	9
	Natural waterbodies	33	21
Type of toilet	Pit latrine	110	92
	Flush toilet	5	4
	Both pit latrine and flush toilets	5	4
Toilet seat conditions	Toilet seat with lid (close without leaving a gap)	47	52
	Toilet seat without lids (close and leaves a gap)	43	48
Hand washing facilities	Yes	89	74
	No	31	26
Waste disposal strategy	Dumping within the household vicinity	119	99
	Use of municipal waste disposal services	1	1

The primary source of water for the majority of participating households (*n* = 95, 62%) was communal taps. Municipal mobile water supply served a considerably smaller proportion of households (*n* = 14, 9%) as their main water source. Borehole ownership within the property was another, but less frequent, water source, reported by only a small number of households (*n* = 12, 8%). Notably, a distinct subset of households (*n* = 33, 21%) relied on natural water sources such as rivers, streams and springs for their primary water needs. Nearly all households (99%) reported relying on on-site waste disposal methods within their own premises. Conversely, a very small percentage of households (1%) utilised municipal waste collection services.

All households in the community have access to toilet facilities, with the majority (92%) utilising pit latrine toilets. A small proportion (4%) use flush toilets, while 4% have both pit latrines and flush toilets. Across the six villages surveyed, pit latrine toilets were predominant, utilised by 92% of households. Flush toilets were less common, used by 4% of households and 4% had both types of toilets. Regarding toilet seat conditions, 60% of households had seats with lids, while 40% did not. Approximately half of the households (52%) reported gaps between the toilet lids and seats when closed, while 48% did not have such gaps. At least 85 (76%) households reported having handwashing facilities with soap. The study revealed a dominant practice of storing water in containers within households (93%). Notably, a significant majority (80%) utilised containers with secure lids, minimising potential contamination risks. However, a minority (20%) lacked lids or had lids with gaps.

Analysis of waste disposal practices indicated that only 46 households (38%) possessed lidded trash cans that were effectively closed. The remaining 74 households (62%) lacked lids or had lids with gaps, potentially contributing to odour and pest issues. In addition, lining trash cans with refuse plastics was not universally practised. Motsinoni and Makaba had the lowest adherence (30% and 20%, respectively), while Mohlakong demonstrated the highest (95%). The study observed varying patterns of outdoor waste accumulation across villages. While 35 households (29%) had no outdoor waste, 72 (60%) had a limited amount and 13 (11%) displayed significant accumulation. Interestingly, no statistically significant association was found between the amount of outdoor waste and waste disposal strategies, emptying frequency or tying trash bags before disposal. Table 2 summarises waste management, sanitation practices and hygiene behaviours utilised by participating households as well as self-reported incidence of diarrhoeal illness in the past 12 months.

**TABLE 2:** Hygiene practices and self-reported incidence of diarrhoeal illness in the past 12 months.

Variables	Category	*n*	%
Temporary store water in containers equipped with lids	Yes	112	93
	No	8	7
Temporary store waste in lidded trash cans	Yes	50	42
	No	70	58
Frequency of waste disposal	Daily	16	13
	When necessary	53	44
	When full	51	43
Amount of outdoor waste	None	35	29
	Few	72	60
	Many	13	11
Toilet lid closure practices	Never	39	32
	Sometimes	44	37
	Always	37	31
Frequency of washing hands - after toilet use	Never	9	8
	Sometimes	30	25
	Always	81	67
Self-reported diarrhoea in the past 12 months	Never	40	33
	Sometimes	73	61
	Always	7	6
Prevalence of diarrhoea per age group	0–5 years	31	22
	6–18 years	41	30
	19 years old and above	67	48

Across different villages, there was diversity in the habit of closing toilet seats: in Motsinoni, 40% of households consistently closed the toilet seat, while in Makaba, only 15% adhered to this practice, with 80% leaving the seat open habitually. Mohlakong displayed a similar range, with 25% consistently closing the toilet seat. Examining the availability of in-house handwashing facilities with soap across villages, Motsinoni and Makaba each had 19 households (21%) lacking this essential amenity. In Mohlakong, 12% of households faced a similar challenge, while in Moshakga and Ramphenyang, 16% and 14% respectively, did not have access to such facilities. Similarly, Mokwasele reported 16% of households without soap-equipped handwashing facilities.

The study found a significant disparity in the prevalence of diarrhoeal episodes that occurred within 12 months before conducting the study. A substantial portion (40 households, 33%) reported no occurrences within the 12 months before the study. Eighty-six per cent of households experienced at least one diarrhoeal episode within the period of 12 months prior to the date of the study. Among households reporting diarrhoeal episodes, the highest proportion (22%, *n* = 31) occurred in adults aged 19 years and older. Children aged 6–18 and 0–5 years accounted for a smaller proportion (30%, *n* = 41 and 48%, *n* = 67, respectively). The analysis revealed village-level variations in the incidence of childhood diarrhoea (0–5 years). Makaba had the highest rate (31%), followed by Mohlakong (32%), Mokwasele (30%) and Ramphenyang (15%). The study did not identify any self-reported cases of diarrhoea within the Moshakga community during the data collection period. Table 3 outlines the historical relationship between experiencing diarrhoea and water storage practices, handwashing facilities, as well as post-toilet handwashing habits across different households in different villages.

**TABLE 3:** Diarrhoeal episodes against other variables.

Variables	Category						*p*-value
	Never		Sometimes		Always		
	*n*	%	*n*	%	*n*	%	
**Storage of water in containers**	-	-	-	-	-	-	< 0.001
Yes	39	97.5	69	94.5	4	57.1	-
No	1	2.5	4	5.5	3	42.9	-
**Have handwashing facilities with soap**	-	-	-	-	-	-	< 0.01
Yes	33	82.5	54	74.0	2	28.6	-
No	7	17.5	19	26.0	5	71.4	-
**Frequency of washing hands after the use of toilet**	-	-	-	-	-	-	< 0.001
Never	2	5.0	6	8.2	1	14.3	-
Sometimes	1	2.5	28	38.4	1	14.3	-
Always	37	92.5	39	53.4	5	71.4	-

Chi-square tests indicate that households that stored water in containers (*p* ≤ 0.001), had access to handwashing facilities (*p* = 0.01), and practised frequent handwashing with soap and water after toilet use (*p* ≤ 0.001) displayed lower rates of diarrhoeal illness.

## Discussion

Water access in a rural area inside GLM is assessed in this study. While all households possessed sanitation facilities, these were not classified as ‘improved’ based on established criteria (e.g. limited contamination risk). The findings echo existing research on water scarcity in developing nations, as reported by the WHO/UNICEF JMP reports on the lack of access to safely managed drinking water, which can lead to contamination risks or water scarcity (WHO/UNICEF JMP 2021). The GLM reports indicate that 75% of residents live near a water source (within 200 m), but 9.3% lack tap water access, and 30% – 40% of villages rely on weekly tanker truck deliveries (Greater Letaba Municipality 2022). This aligns with the study, where 62% rely on communal taps, suggesting a centralised system for a large portion of the community. However, the dependence on natural water sources (21%) and private boreholes (8%) highlights limitations in existing infrastructure. Previous studies report a significant urban-rural divide in access to safe drinking water, with rural areas often facing limited access. Official statistics from Botakara village in Asia demonstrated this disparity, with a significantly lower proportion (28%) having access to safely managed water compared to urban areas (around 90%) (Omarova et al. 2019). In contrast to other earlier studies conducted in Ethiopia (35% and 37.5%), this study found a decreased dependence on natural water sources (21%) (Atumo Ante, Asefa Bogale & Mohammed Adem 2023; Bogale 2020; Kassie & Hayelom 2017). In order to lower the risk of schistosomiasis transmission, a study was carried out in the rural South African district of uMkhanyakude with the goal of identifying important environmental and psychosocial factors impacting behaviour modification. The results also showed that taps (10.53%) and pump or boreholes (7.02%) were the community’s main sources of drinking water. However, 77.19% of respondents said they relied on surface water sources, including rivers, dams and unprotected dug wells (Mulopo, Kalinda & Chimbari 2020). Public health problems are raised by the distribution of water sources that have been identified. Communal taps, while accessible, can be shared by many and potentially transmit diseases. Municipal mobile water supply may be more reliable, but not universally available. Private boreholes and natural water sources can be contaminated and unreliable. This suggests a portion of the community may lack access to a safe and reliable water supply, which creates favourable conditions for the transmission of infectious diseases, including schistosomiasis.

In various global regions, the practice of storing water in containers is widespread, especially prevalent in areas with limited access to safe and reliable water sources. Within this study, the participation of 112 households (representing 93% of the surveyed population) in storing water within their homes highlights the ubiquity of this practice in the community. This finding resonates with prior research in rural areas of Ethiopia, which reported a similar prevalence of 95.5% among households storing water in covered containers (Kassie & Hayelom 2017). In addition, similar to this study, a separate investigation in Goa, India, reported a high prevalence of containerised water storage among rural households, with 89.0% storing water in closed containers (Gaude & Dessai 2019). The primary motivation behind this practice is likely to ensure continuous water availability, particularly during interruptions in water service because of water scarcity in GLM. Additionally, the homes are far from the communal taps. However, it is crucial to recognise that improperly managed water storage in containers poses significant public health risks.

Containers lacking tightly sealed lids, or worse, without lids altogether, are more susceptible to contamination. Such openings increase the likelihood of contaminants such as dirt, pathogens and insects (e.g. cockroaches) entering the stored water, thereby compromising its quality (Patel et al. 2022). The current study reveals that a majority of households utilise water containers equipped with properly fitting lids, which helps mitigate the risk of contamination. Previous studies have similarly expressed concerns about water quality in stored containers. For instance, a study in rural Ethiopia found that open containers used for water storage were contaminated with faecal coliforms (Berihun et al. 2023). In contrast, the present study identifies 24 households (20% of the sample) lacking adequately sealed lids on their water containers, thereby increasing the potential for contamination. Research conducted in the hilly rural areas of mid and far-Western Nepal indicated that 32% of households did not use lids to cover their water storage containers (Gaffan et al. 2022). This prevalence in the current study is lower than reported in certain other studies. Nonetheless, even a minority of households lacking proper lids is concerning, as it elevates the risk of waterborne illness within the community.

In the community examined in this study, every household reported access to a pit latrine toilet facility, surpassing conditions found in many developing nations. Hygienic sanitation facilities play a crucial role in public health. Benin found that nearly two-thirds of households (66.4%) used unimproved sanitation options (Gaffan et al. 2022). As of 2022, the global sanitation crisis continues to affect 3.5 billion people. Only between 36% and 46% of the world’s rural population had access to safely managed sanitation services, which are defined as improved, non-shared facilities ensuring safe excreta treatment or disposal (UNICEF & WHO 2023). Despite this, an estimated 419 million people still practice open defecation, highlighting ongoing challenges in sanitation access worldwide (UNICEF & WHO 2023). These studies indicate that a significant proportion of rural populations still rely on pit latrines.

Recent research also reveals disparities in latrine coverage across different regions: approximately 71.8% of households in north-eastern Ethiopia, 55% in north-western Ethiopia and 85% in rural Kenya utilise pit latrines (Asnake & Adane 2020; Kassie & Hayelom 2017). Despite these improvements, a significant proportion of households in the current study (about 52%) reported issues with inadequate sealing of toilet lids after use. Poor sanitation conditions and the presence of dirt provide ideal conditions for pest infestations, as these pests find food, water and shelter readily available (Abudin, Martini & Nurjazuli 2023; Gondhalekar et al. 2021; Novia & Windusari 2024). According to GLM reports, approximately 72.6% of residents engage in individual waste disposal practices within the municipal jurisdiction (Greater Letaba Municipality 2022). This aligns with the study findings, where 99% of households dispose of waste within their premises, while only 1% utilise municipal waste services. Waste management activities in the area are made more difficult by obstacles, including small roads and a lack of room for huge trash cans.

Irrespective of the type of toilet facility used, 31% of the sample consistently closed the toilet lid when not in use. In contrast, 37% of participants, totalling 44 individuals, occasionally closed the toilet lid. Similar findings were found in a Hong Kong study on toilet cleanliness habits, where 43.9% of participants never covered the toilet lid before flushing (Wu et al. 2019). Factors contributing to inconsistent lid closure in the current study include a lack of awareness about the benefits, forgetfulness, convenience and cultural habits. The absence of toilet seat lids may also influence non-closure behaviour. Both studies emphasise the importance of closing the toilet lid to minimise the spread of bacteria and potential transmission of diseases.

While access to hand hygiene facilities is high in the study, WHO/UNICEF JMP also highlight an estimation of 2 billion of the world’s population still lacking access to basic household handwashing supplies, such as soap and water (United Nations 2023). The study found that 68% of households reported consistent handwashing with soap and water after using the toilet, which does not align with findings from other studies. The highest proportions of individuals who rarely or never wash their hands after using the restroom were reported in Tuvalu (17.7%), Mauritania (24.4%) and East Timor (27.5%). Overall, a notably high prevalence of this behaviour was observed across many countries in Africa (Smith et al. 2021). In addition, Sitotaw, Melkie and Temesgen (2021) also reported a lower proportion (35%) of participants who did not practice handwashing after toilet use, highlighting variability in hygiene practices across different studies. The Chi-square test results with a p-value of 0.040 indicate a statistically significant difference in the frequency of handwashing with soap and water after toilet use, depending on the availability of handwashing facilities. Specifically, 68% of individuals are more inclined to wash their hands after using the toilet if handwashing facilities are readily available. This suggests that accessibility and visibility of handwashing facilities play a crucial role in promoting hygienic practices. In addition, various studies agree that improvements in water supply, sanitation and hygiene have been shown to reduce waterborne diseases, such as diarrhoea (Smith et al. 2021; Wolf et al. 2023; World Health Organization 2022). In the surveyed households, 67% reported experiencing at least one case of diarrhoea in the past year, indicating variability in vulnerability among households. Notably, 22% of households with diarrhoea cases had children aged 5 years and below, consistent with the susceptibility of young children to diarrhoeal illnesses because of their developing immune systems. Similar findings were observed by Daniel et al. (2020) and others who reported a 5% incidence of diarrhoea among children under 5 years in their study. The study also found significant associations between water storage practices, availability of handwashing facilities, handwashing behaviour and reduced risk of diarrhoea (*p* ≤ 0.001). Household members who stored water in containers, had access to handwashing facilities, and consistently washed hands with soap and water after using the toilet were significantly less likely to develop diarrhoea compared to those who did not adhere to these practices. These findings underscore the importance of these hygiene practices in preventing diarrhoeal diseases within households.

### Strength and limitations

The study identified significant correlations between water storage practices, the availability of handwashing facilities, handwashing behaviours and the incidence of diarrhoeal diseases. Moreover, it underscores the critical need for more advanced drinking water sources to reduce reliance on natural water bodies and improve waste management systems, as well as adequate sanitation. These findings will be instrumental for researchers, policymakers, government bodies, municipalities and educational institutions in shaping policies, programmes and strategies to address sanitation, hygiene and waste management challenges. Such interventions are essential for disease prevention and for creating healthier, safer living conditions in developing countries. A key limitation of the study is that it could not determine whether specific WASH practices caused the diarrhoeal illnesses reported by households in the 12 months leading up to the research. The study did not collect data on the distance travelled between households and water collection points or sanitation facilities. Additionally, the study did not involve physical observations to assess the availability and condition of handwashing facilities.

### Recommendations

Governments, municipalities and relevant stakeholders should strengthen sector-wide investment and capacity building by prioritising initiatives such as the adoption of water conservation technologies, the promotion of innovation and evidence-based practices and the enhancement of cross-sectoral coordination and collaboration. Furthermore, a more integrated and holistic approach should be adopted to ensure universal access to clean drinking water and properly maintained sanitation facilities, such as ventilated toilets with secure lids and functional handwashing stations. These efforts are critical to advancing the effective implementation of the SDGs by 2030. In addition, community waste management plans should be implemented to provide households with municipal waste collection services and encourage sustainable practices such as recycling and composting. Furthermore, education initiatives in healthcare and training institutions should promote sanitation and hygiene, emphasising personal hygiene practices such as closing toilet lids, washing hands and maintaining clutter-free environments. Lastly, WASH strategies should encourage positive behavioural practices and foster public commitment in order to ensure good health and well-being in the communities.

## Conclusion

Safe drinking water and adequate sanitation infrastructure are essential for promoting individual and community well-being, including public health and a dignified quality of life. The study highlights the ongoing necessity for more advanced drinking water sources to reduce dependence on natural water bodies and to enhance waste management services. While the study indicates satisfactory provision of toilet facilities, there is a notable need for infrastructure improvements and toilet design modifications to facilitate proper closure of toilet seat lids. Furthermore, promoting good personal hygiene practices, such as closing toilet lids after use, thorough handwashing with soap and water after using the toilet and before handling food, and maintaining a clean environment, is essential. These practices are crucial for reducing the incidence of diarrhoeal illnesses and improving overall health outcomes.
